# Association between systemic rheumatic diseases and dementia risk: A meta-analysis

**DOI:** 10.3389/fimmu.2022.1054246

**Published:** 2022-11-09

**Authors:** Yao-Chin Wang, Muh-Shi Lin, Abel Po-Hao Huang, Chieh-Chen Wu, Woon-Man Kung

**Affiliations:** ^1^ Department of Emergency, Min-Sheng General Hospital, Taoyuan, Taiwan; ^2^ Graduate Institute of Injury Prevention and Control, College of Public Health, Taipei Medical University, Taipei, Taiwan; ^3^ Division of Neurosurgery, Department of Surgery, Kuang Tien General Hospital, Taichung, Taiwan; ^4^ Department of Biotechnology and Animal Science, College of Bioresources, National Ilan University, Yilan, Taiwan; ^5^ Department of Biotechnology, College of Medical and Health Care, Hung Kuang University, Taichung, Taiwan; ^6^ Department of Health Business Administration, College of Medical and Health Care, Hung Kuang University, Taichung, Taiwan; ^7^ Department of Surgery, Division of Neurosurgery, National Taiwan University Hospital, College of Medicine, National Taiwan University, Taipei, Taiwan; ^8^ Department of Healthcare Information and Management, School of Health Technology, Ming Chuan University, Taipei, Taiwan; ^9^ Division of Neurosurgery, Department of Surgery, Taipei Tzu Chi Hospital, Buddhist Tzu Chi Medical Foundation, New Taipei City, Taiwan; ^10^ Department of Exercise and Health Promotion, College of Kinesiology and Health, Chinese Culture University, Taipei, Taiwan

**Keywords:** systemic rheumatic diseases, osteoarthritis, rheumatoid arthritis, systemic lupus erythematosus, Sjogren’s syndrome, dementia

## Abstract

**Background and aims:**

Epidemiological studies have been conducted on the relationship between systemic rheumatic diseases (SRDs) and dementia. Therefore, we focused on determining the extent of alliances bounded by SRDs, along with the risk of dementia.

**Materials and methods:**

Two independent reviewers assessed all studies retrieved from the PubMed, EMBASE, Scopus, and Web of Science databases between January 1, 2000 and November 30, 2021. Only observational studies that estimated the possibility of dementia in participants with SRD were considered. The random-effects model was applied to forecast pooled risk ratios (RRs) and 95% confidence intervals (CI). Heterogeneity among the studies was evaluated using the Q and I^2^ statistics. The quality of the included studies was assessed using the Newcastle-Ottawa Scale. Funnel plots were used to calculate the risk of bias.

**Results:**

Seventeen observational studies with 17,717,473 participants were recruited. Our findings showed that among the participants with SRDs, those with osteoarthritis, systemic lupus erythematosus, and Sjogren’s syndrome were highly related to an elevated risk of dementia (pooled RR: 1.31; 95% CI: 1.15–1.49, p<0.001; pooled RR: 1.43; 95% CI: 1.19–1.73, p<0.001; and pooled RR: 1.26; 95% CI: 1.14–1.39, p<0.001, respectively). However, participants with rheumatoid arthritis (RA) were not associated with an increased risk of dementia (pooled RR: 0.98; 95% CI: 0.90–1.07, p<0.001).

**Conclusion:**

This systematic review and meta-analysis demonstrated an increased dementia risk among SRDs participants, except for RA.

## Introduction

Dementia is a common neurological disorder associated with impaired performance in daily activities (1, 2). The total number of dementia victims is forecast to increase to 78,000,000 by 2030 and 139,000,000 by 2050 (3, 4). Alzheimer’s disease (AD) is the most commonly seen dementia in the aging population, contributing roughly 60–70% of all dementia cases (5, 6). In contrast, the remaining dementia categories consist of dementia with Lewy bodies, vascular dementia, and frontotemporal dementia (7). Previous studies, mainly based on histopathological examination, have shown that these two biomarkers (neuritic plaques and neurofibrillary tangles caused by glial activation) are associated with dementia (8, 9). Multiple evidence-based medicine studies have reported that inflammation plays an important role in AD pathogenesis (10, 11). However, no medication is available to reverse or prevent the progression of AD (12).

Systemic rheumatic diseases (SRDs) such as osteoarthritis (OA), rheumatoid arthritis (RA), systemic lupus erythematosus (SLE), and Sjogren’s syndrome (SS) are chronic inflammatory conditions that are common causes of disability (13). Over the decades, the prevalence of SRDs has increased and evidence shows that SRDs are associated with substantial morbidity and mortality (14, 15). Several epidemiological investigations have found a relationship between SRDs and the risk of dementia. However, the findings from previous studies remain controversial, and some methodological limitations have been observed. Previous biological studies have demonstrated that these two diseases share similar inflammatory mechanisms. Indeed, pro-inflammatory cytokines [interleukin (IL)-1b, IL-6, and tumor necrosis factor (TNF)-α] are linked to an increased possibility of dementia and are also associated with the pathogenesis (16–18).

Regarding the current evidence of SRDs, little information can be gathered if SRDs are related to dementia. Thus, we examined the relationship between SRDs and dementia risk through an updated systematic review of evidence (19).

## Materials and methods

The Preferred Reporting Items for Systematic Reviews and Meta-analyses (20) guidelines were used to prepare this meta-analysis (2, 20).

### Data sources and literature search

An extensive search was conducted in the PubMed, MEDLINE, Embase, and Web of Science databases for studies evaluating the association of dementia with SRDs and dementia risk. A search strategy was developed based on the PICO [Patient, Intervention/Exposure, Comparison, Outcome] questions, and two expert authors (Y.-C.W., M.-S.L.) assisted in developing the search strategies, which are provided in **Supplementary Table S1**. Articles published in English were considered in this study, although geographic location was not imposed. In addition, the citation directories of the included references and relevant review articles on this topic were screened for additional studies.

### Study selection and outcomes

To analyze the possibility of dementia incidence and diminish potential bias, our primary target included only randomized controlled trials (RCTs). However, no RCTs have been published on this topic. Therefore, we included observational studies (e.g., case-control and cohort studies) to reduce the recall bias. Our inclusion criteria were as follows: (1) all observational studies that evaluated the association between dementia and SRDs, (2) provided accurate information about their methodology, and (3) reported the risk estimates of dementia. We considered studies with proven diagnoses of dementia and SRDs, based on clinical guidelines or standard diagnostic codes. If studies were published as cross-sectional, case reports, editorials, review articles, or non-human studies, they were excluded. The same two expert authors (Y.-C.W. and M.-S.L.) independently examined all the topics and abstracts of the retrieved manuscripts using predefined criteria. Subsequently, the entire content of possible articles was screened for acceptability. Any differences during the study screening were resolved through discussion with the principal investigator until a final decision was made.

### Data extraction and risk of bias assessment

The same expert authors (Y.-C.W., M.-S.L.) separately extracted the following data: author name (first author only), publication year, origin, database, population characteristics, study period, participant selection process, adjusting factors (drugs, diseases etc.), number of participants, and outcome of interest (dementia). We collected only data on adjusted risk estimates, including risk ratios (RRs), odds ratios (ORs), and hazard ratios (HRs) with 95% confidence intervals (CIs). To examine the risk of bias for the included articles, the Newcastle-Ottawa Scale was applied by the same expert authors. All controversies within the period of data extraction and the possibility of bias calculation were finalized through a dissertation with other authors.

### Statistical analyses

We conducted a comprehensive meta-analysis (CMA) of our study. We calculated the overall RRs of dementia and utilized the HRs and ORs from the included studies. Pooled RRs with corresponding CIs were analyzed using a random-effects model to reduce considerable clinical heterogeneity. The I^2^ statistic was used to evaluate appropriate heterogeneity between studies. I^2^ statistics ranging from 0–25%, 25–50%, 50–75%, and 75–100% were expressed as very low, low, medium, and high heterogeneity, respectively (21, 22). We also performed subgroup analyses based on study design, location, and disease to determine whether other factors affected the findings. We also presented funnel plots to calculate publication bias. A *p-*value of <0.05 was considered significant.

## Results

### Study selection

**Figure 1:**
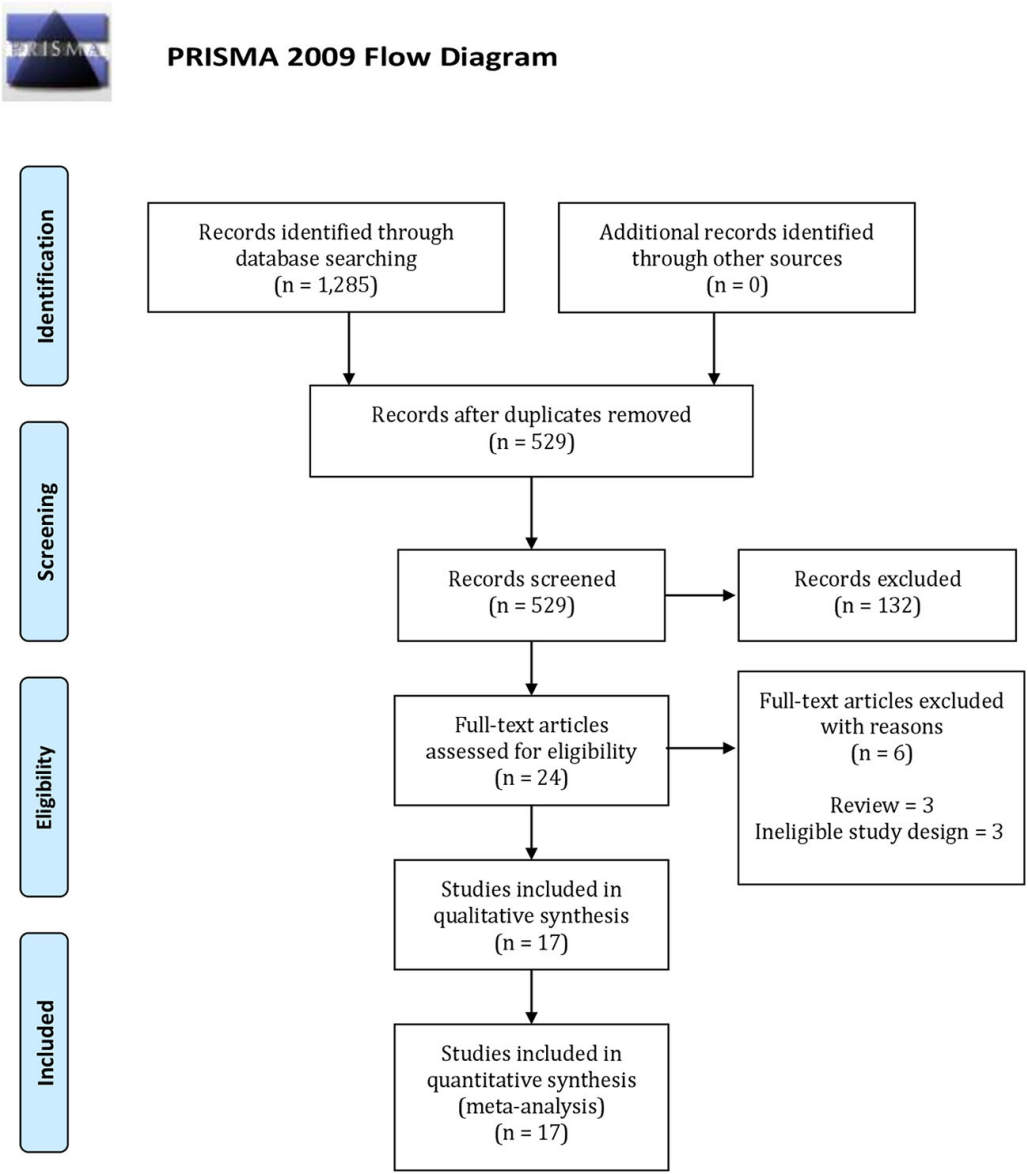
Preferred reporting items for systematic reviews and meta-analyses based search strategy flow chart.

The PRISMA selection flowchart is presented in **Figure 1**. Initially, 1,285 articles were selected from the database. Subsequent screening of all titles and abstracts revealed 24 full-text articles for the initial study qualification. Finally, 17 studies were included in the current research (23–39).

### Characteristics of included studies

The basic characteristics of the results and important outcomes are outlined in **Table 1**. Seventeen observational studies (case-control and cohort) with 17,717,473 participants were included. In total, 12 studies were conducted in Asia, three in Europe, and two in North America. The publication period ranged from 2010 (37) to 2021 (24). SRDs and dementia were illustrated by *the International Classification of Diseases (ICD) Ninth and Tenth Revision*. The modified covariates of all encompassed studies are presented in **Supplementary Table S2**.

**Table 1:** Baseline characteristics of included studies.

Source	Data source	No. of cases/controls	Study period	Age, mean (SD), y	Female sex, %	Definition of SRDs	Risk of dementia
Swain 2021	CPRD	209,602/208799	1997-2017	61.1	58	Clinical codes	OA: 1.62 (1.56-1.68)
		221807/221807	1997-2017	61.1 (13.2)	58	Clinical codes	OA: 1.09 (0.99, 1.19)
Park 2021	KNHIS	6,028/30,140	2002-2013	Range	70	ICD-10	RA: 0.96 (0.83–1.11)
							SS: 1.1 (0.88–1.38)
							SLE: 2.48 (1.19–5.15)
Innes-2020	Medicare	4,545/12,389	2001/2013	75.90 (0.11)	67.2	ICD-9	OA: 1.23 (1.06-1.42)
Min 2020	KNHIS-NSC	11,443/45,772	2002-2013	Range	68	ICD-10	RA: 0.96 (0.78-1.16)
	KNHIS-NSC	4420/17,680	2002-2013	Range	78.1	ICD-10	RA: 0.91 (0.76-1.10)
Huang 2019	TNHIRD	20,707/62,121	2000-2005	53.0 (14.5)	72.2	ICD-9	RA: 0.63 (0.55-0.72)
Chen 2019	TLHID	4,063/81,273	2000-2012	63.7 (9.4)	74.6	ICD-9	SS: 1.21 (1.02-1.45)
Chen 2018	TNHIRD	10108/61080	2000-2010	73.2 (9.6)	50.64	ICD-9	RA: 1.11 (1.10–1.22)
							SS: 1.28 (1.11–1.47)
							SLE: 1.55 (1.16–2.07)
							OA: 1.47 (1.40–1.54)
Li 2018	Swedish Nationwide database	236,278/ 14,175,00	1964-2010	55.6	49.4	ICD-7-10	RA: 1.05 (1.00-1.11)
							SS: 1.48 (1.22-1.78)
							SLE: 1.15 (0.91-1.44)
Wang 2018	TNHIRD	2618/5236	2001-2011	76.1	59	ICD-9	OA: 1.23 (1.07–1.41)
Lin 2018	TNHIRD	34,600/138,640	2001-2012	59.8	77	ICD-9	RA: 1.14 (1.06–1.32)
							SLE: 1.07 (0.86–1.34)
							SS: 1.46 (1.32–1.63)
Kao 2016	TLHID	2271/6,813	2001-2013	76.5 (8.2)	61.1	ICD-9	RA: 0.73 (0.55-0.98)
Lin 2016	NHIR	1,074/5,370	2004-2010	Range	86.78	ICD-9	SLE: 2.14 (1.26-3.63)
Huang 2015	TLHID	35,149/70,298	2004-2007	Range	63.2	ICD-9	OA: 1.25 (1.10–1.43)
Lu 2014	TLHID	1221/6,105	2000-2005	Range	79.4	ICD-9	RA: 1.20 (0.75–1.92)
							SLE: 2.86 (1.16–7.05)
							SS: 0.81 (0.33–1.99)
Veeranki 2017	MHAS	605/2,076	2001-2012	66.5 (5.4)	56.8	Clinical code	RA: 1.23 (0.86–1.76)
Wotton 2017	ENHES	1,833,827*	1999-2012	NR	NR	ICD-9-10	RA: 1.13 (1.11-1.15)
							SS: 1.14 (1.04-1.24)
							SLE: 1.46 (1.32-1.61)
Kang 2010	NHIRD	1,974/9,870	2006-2007	49.7 (21)	76.2	ICD-9	SS: 1.13 (0.76–1.70)

CPRD, Clinical Practice Research Datalink; KNISCD, Korean National Health Insurance Service; KNISCD-NSC, Korean National Health Insurance Service-National Sample Cohort; TLHID, Taiwan Longitudinal Health Insurance Database; TNHIRD, Taiwan National Health Insurance Research Database; CAIDE, Cardiovascular risk factors, Aging and Incidence of Dementia; ENHES, England National Hospital Episode Statistics; * = Total number of patients; NR = Not reported.

### Risk of bias assessment

NOS assessment was used to examine risk bias in the selected studies. The methodological study quality was divided into two categories (high, >7; low, ≤7) according to the NOS value. In total, 11 studies had low risk bias, five had moderate risk bias, and one had high risk bias. However, the general quality of evidence was high in the meta-analysis (**Supplementary Table S2**).

### Risk for dementia

**Figure 2:**
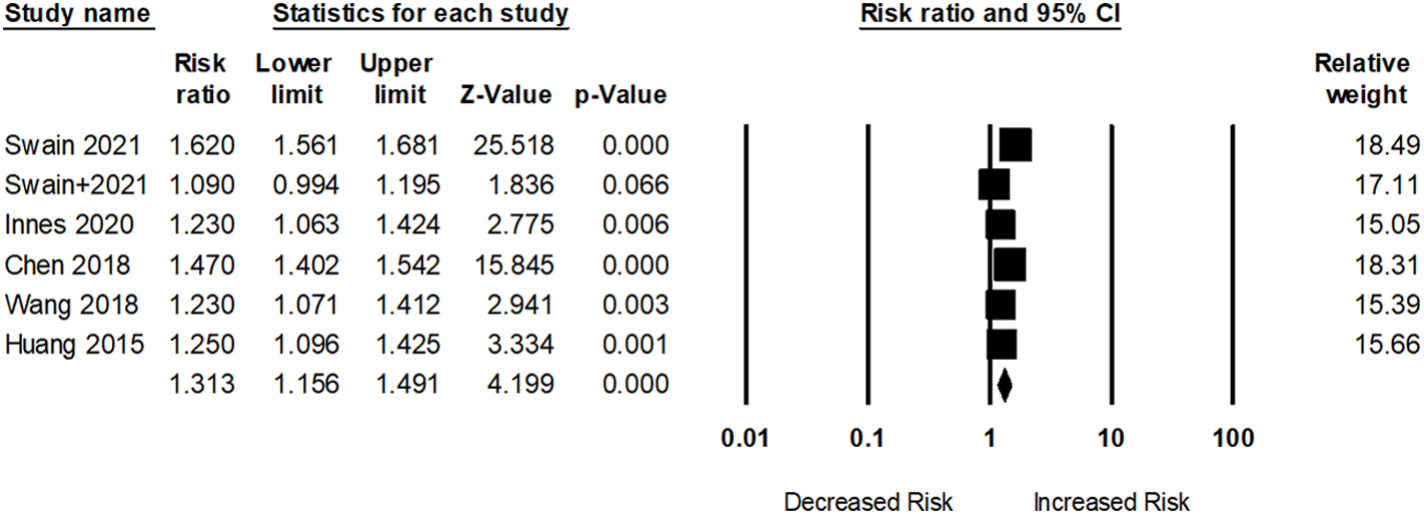
Association between osteoarthritis (OA) and dementia risk. + indicates that the same author has 2 studies.

Sum of 17 researches with 17,717,473 participants examined the association between dementia risk and SRDs. However, five studies assessed dementia risk within OA participants. As shown in **Figure 2**, participants with OA had an elevated dementia risk (pooled RR: 1.31; 95% CI: 1.15–1.49) compared to those without OA. The I^2^ statistic of these studies was 94.10%, indicating a high risk of heterogeneity.

**Figure 3:**
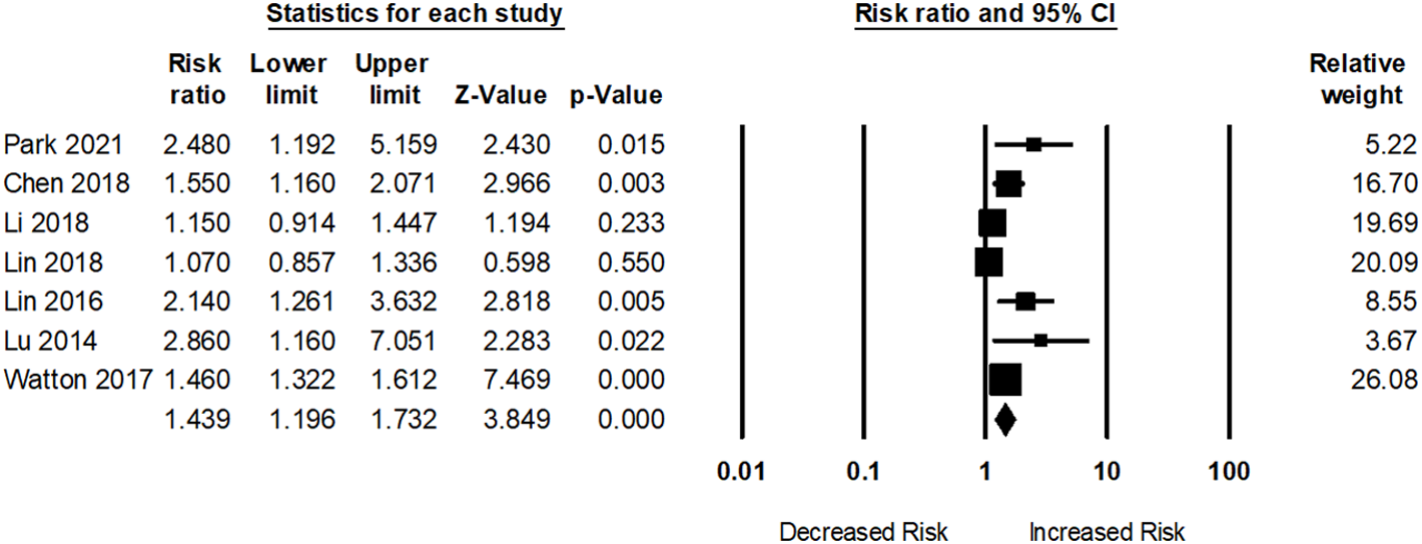
Association between systemic lupus erythematosus (SLE) and dementia risk.

Seven studies assessed dementia risk in participants with SLE. As presented in **Figure 3**, participants with SLE had an elevated dementia risk (pooled RR: 1.43; 95% CI: 1.19–1.73) compared to those without SLE. The I^2^ statistic among these studies was 64.38%, indicating a moderate risk of heterogeneity.

**Figure 4:**
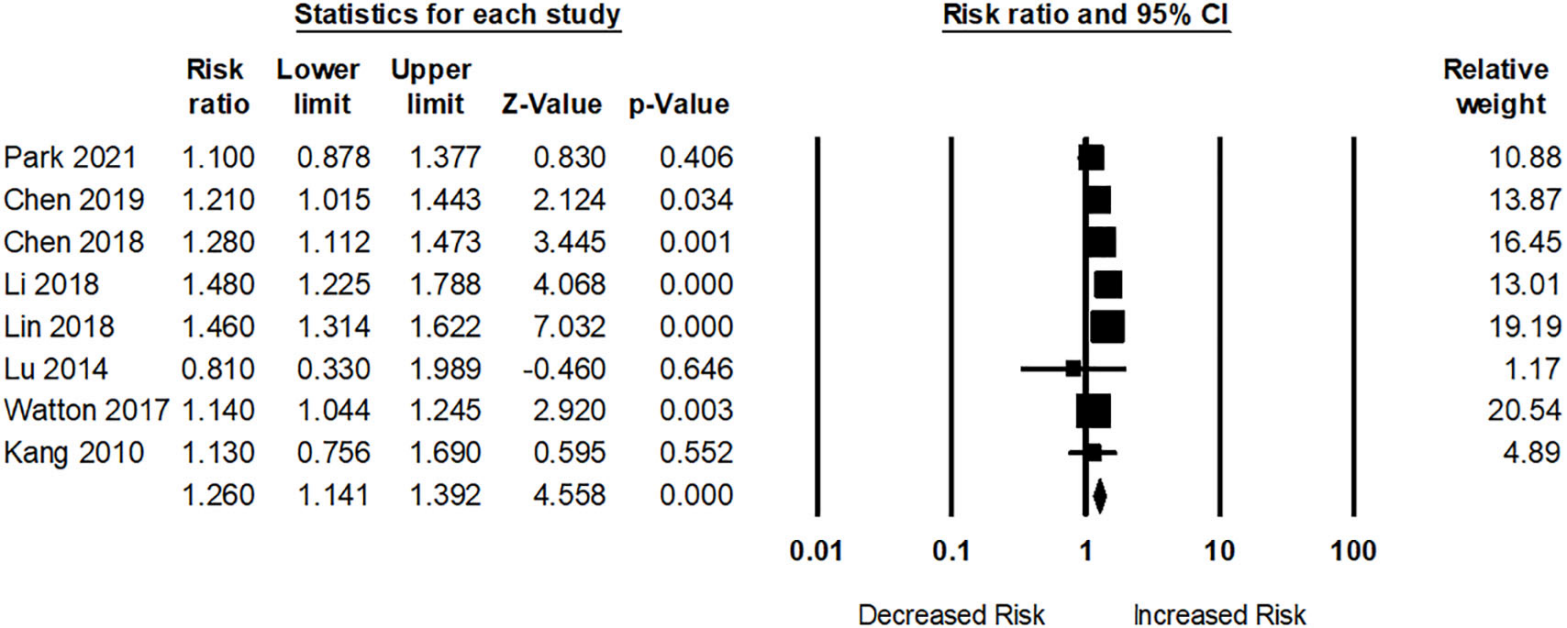
Association between Sjogren’s syndrome (SS) and dementia risk.

Eight studies evaluated dementia risk among participants with SS. Participants with SS were associated with a significantly greater risk of dementia (pooled RR: 1.26; 95% CI: 1.14–1.39) compared to those without SS (**Figure 4**). The I^2^ statistics of these studies were 61.36%, indicating a moderate heterogeneity risk.

**Figure 5:**
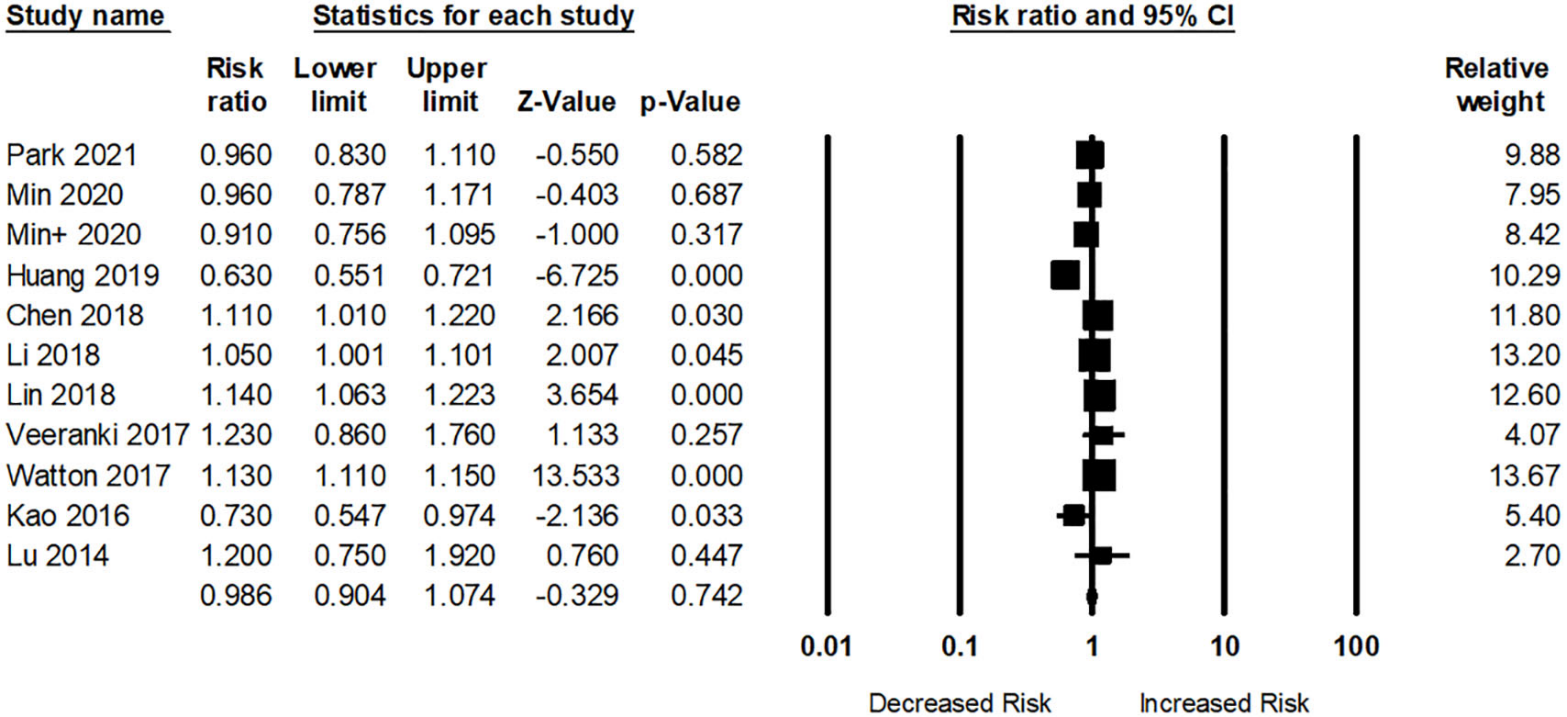
Association between rheumatoid arthritis (RA) and dementia risk. + indicates that the same author has 2 studies.

In total, ten studies examined the risk of dementia among participants with RA. Participants with RA were not related to any dementia risk (pooled RR: 0.98; 95% CI: 0.90–1.07) compared to those without RA (**Figure 5**). The I^2^ statistic among these studies was 89.61%, indicating a high risk of heterogeneity.

### Subgroup analyses

**Table 2** shows the results of subgroup analyses. Two studies assessed the risk of dementia in participants aged >65 years with SLE. For participants aged <65 years, the adjusted pooled RR was 1.93; 95% CI: 0.31–11.75 and the heterogeneity of the studies was high (I^2^ = 81.10, Q = 5.29, tau^2^ = 1.10). For participants aged <65 years, the adjusted pooled RR was 1.23; 95% CI: 0.96–1.56 and the heterogeneity in these studies was very low (I^2^ = 0, Q = 0.87, tau^2^ = 0). However, the risk of dementia among SLE patients was higher in female in comparison with male (RR: 1.47; 95% CI: 1.32–1.64 vs RR: 1.36; 95% CI: 1.04–1.75.

**Table 2:** Subgroup analysis.

			Effect size			Heterogeneity		
Subgroup	Class	Study	RR	95%CI	*p*	Q	I^2^	τ^2^
SLE	Overall	7	1.43	1.19-1.73	<0.001	16.84	64.38	0.03
	< 65 year	2	1.93	0.31-11.75	0.47	5.29	81.10	1.40
	> 65 year	2	1.23	0.96-1.56	0.09	0.87	0	0
	Male	1	1.36	1.04-1.75	0.05	–	–	–
	Female	1	1.47	1.32-1.64	0.05	–	–	–
	AD	2	1.37	0.60-3.14	0.45	4.40	77.29	0.28
	VaD	2	2.29	0.93-5.63	0.06	2.52	60.40	0.29
SS	Overall	8	1.26	1.14-1.39	<0.001	18.11	61.36	0.01
	< 65 year	1	1.57	1.24-1.98	0.05	–	–	–
	> 65 year	1	1.45	1.30-1.63	0.05	–	–	–
	Male	2	1.24	1.06-.1.45	0.005	0.44	0	0
	Female	1	1.11	1.02-1.22	0.05	–	–	–
RA	Overall	10	0.98	0.90-1.07	0.74	96.30	89.61	0.01
	< 65 year	3	0.87	0.60-1.27	0.48	12.55	76.09	0.10
	> 65 year	3	0.79	0.54-1.14	0.21	60.91	95.07	0.13
	Male	4	0.77	0.51-1.17	0.22	43.98	90.90	0.18
	Female	4	0.86	0.67-1.10	0.23	44.34	90.98	0.06

RA, Rheumatoid arthritis; SS, Sjogren’s syndrome; SLE, Systemic lupus erythematosus; AD, Alzheimer’s disease; VaD, Vascular dementia.

The dementia risk was slightly higher in female than male participants with RA (RR: 0.86 vs RR: 0.77). However, the risk of excessive dementia was significantly higher in male than females among SS participants (RR: 1.24 vs RR: 1.11).

### Risk of bias

**Figure 6:**
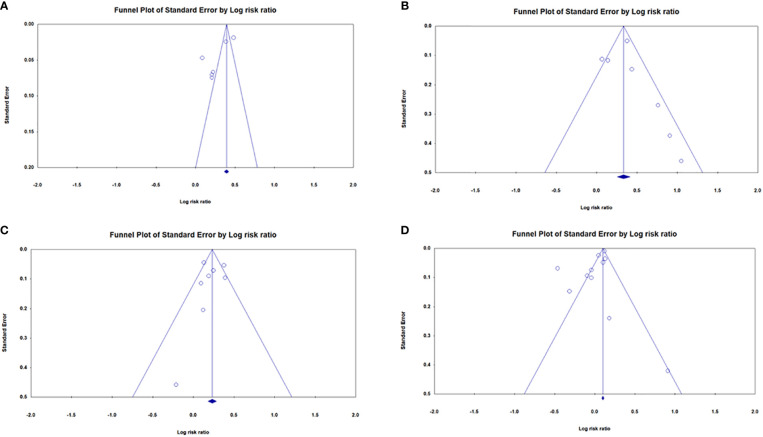
Funnel plots for the association between dementia risk and **(A)** OA, **(B)** SLE, **(C)** SS, and **(D)** RA.

**Figure 6** illustrates the funnel plots for publication bias. **Figure 6A** shows a funnel plot that demonstrates the publication bias of the studies. However, **Figures 6B–D** showed no publication bias. Egger’s regression test was used to calculate funnel asymmetry, which indicated publication bias (p<0.03).

**Figure 7:**
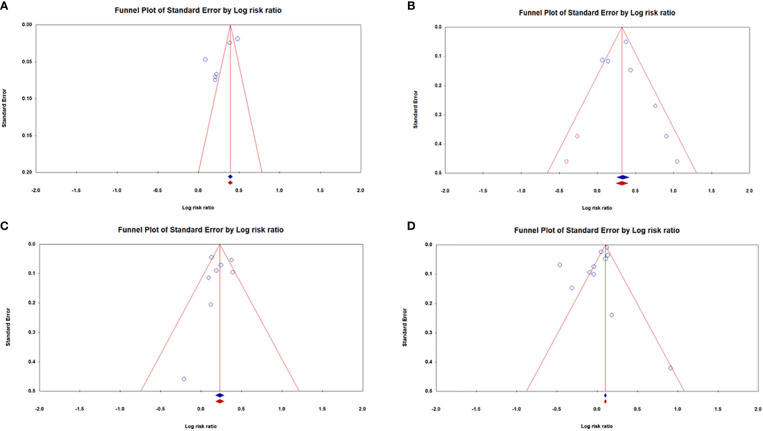
Funnel plots for the association between dementia risk and **(A)** OA, **(B)** SLE, **(C)** SS, and **(D)** RA after using a trim-and-fill method to diminish the consequences of existing publication bias.

**Figure 7** illustrates funnel plots for publication bias after applying the trim-and-fill method. **Figures 7A–D** shows funnel plots with missing research inserted using the trim-and-fill method. There was no missing data imputed in the plots. The overall log risk ratios of **Figures 7A-D** were: 1.31; 95% CI: 1.15–1.49, 1.36; 95% CI: 1.12–1.64, 1.26; 95% CI: 1.14–1.39, and 0.98; 95% CI: 0.90–1.07, respectively.

## Discussion

### Main findings

To the best of our knowledge, this is the first meta-analysis examining the association between SRDs and dementia. An analysis based on 17 observational studies showed that SRDs, such as OA, SLE, and SS, were associated with an increased risk of dementia. However, no relationship was found between RA and the risk of dementia.

The relationship between chronic inflammation and dementia is well established. SRDs such as OA, SLE, and SS, which trigger systemic or organ-specific inflammation, can induce cognitive impairment (30, 36). There is no clear evidence of a pertinent mechanism between them. Several possibilities may help explain the biological mechanism because SRDs and dementia share common risk factors. Previous biological studies have reported that neuroinflammation, a prominent hallmark of dementia, is triggered by microglial cell activation (40–43). Activated microglial cells are further divided into M1 (classical phenotype) and M2 (alternatively activated phenotype) phenotypes. The M1 phenotype releases overwhelming amounts of inflammatory cytokines such as IL-1β, IL-6, IL-11, IL-12, and TNF-α, which may cause cell apoptosis and ultimately lead to loss of neurons (44–46). In addition, the M2 phenotype increases the production of pro-inflammatory cytokines [IL-4, interferon-gamma-inducible protein (IP)-10] and reactive oxygen species (ROS) (47). Recently, the M1/M2 paradigm of microglial cell activation has been evaluated in some neurodegenerative diseases to discover some of the explicit biological mechanisms of immunopathogenesis (48).

Previous studies have also shown that the level of serum IL-1b is extremely high in dementia participants compared to controls (48, 49). Increasing evidence has shown an essential factor for IL-1b overexpression and the deterioration of tau phosphorylation function and tangle formation (50). Moreover, a higher level of IL-1b can hamper the amyloid-beta (Aβ) clearance functions of microglia (51) and increase blood-brain barrier permeability, which is mainly responsible for the accumulation of Aβ in the brain (19, 52, 53). Pro-inflammatory cytokines, such as TNF, IL-1, IL-6, and IL-17 in SRDs may increase the risk of dementia through cell apoptosis and neuronal damage (54). Another reason is the use of anti-inflammatory drugs that are linked to an elevated risk of hypertension and cardiovascular disease, both of which are thought to be the main hallmarks of dementia and cognitive impairment (55). Moreover, the use of glucocorticoids can decrease the volume of the hippocampus and accelerate dementia (56). Recently, an investigation has demonstrated that the use of disease-modifying anti-rheumatic drugs (DMARDs) can increase the risk of dementia (57) because methotrexate may reduce the level of folic acid in the brain (58) and alter the amino acids of the hippocampus (59). Nevertheless, our results showed that RA was not associated with an increased risk of dementia. Meanwhile, earlier researchers have also reported that the use of anti-inflammatory drugs or granulocyte macrophage colony-stimulating factors (GM-CSFs) can decrease the risk of dementia in RA participants (60, 61). An alternative mechanism may affect cognitive function or the risk of dementia in patients with lupus. Several studies have highlighted the association between antiphospholipid autoantibodies and cognitive problems or dementia, including patients with antiphospholipid autoantibodies and lupus (62, 63). RA pathogenesis of RA is the production of anti-cyclic citrullinated peptide antibody (anti-CCP Ab), and periodontitis is a widely known environmental trigger. Moreover, epidemiological studies also showed an association between periodontitis and dementia (40, 64, 65). A previous study reported that non-steroidal anti-inflammatory drugs (NSAIDs) reduced the risk of dementia in collagen-induced arthritis (CIA) by normalizing the activated mitogen-activated protein kinase (MAPK) and nuclear factor kappa B (NF-κB) pathways (66). More biological studies are warranted to determine the relationship between RA and reduction in dementia.

The primary advantage of the present study is the utilization of an extensive search strategy to identify and select studies and to evaluate the risk bias using standard guidelines, thereby minimizing selection bias. An expert researcher (with more than five years of experience in systematic reviews and meta-analyses) and an epidemiologist developed the search strategy, which was further validated by three senior faculty members. Our study had some limitations. First, all the studies used diagnostic codes. Therefore, selection bias might exist. Second, the majority of these studies were published in Asian countries, which might have introduced some geographical bias. Third, no study has reported the duration of SRDs or the risk of dementia. Therefore, we were unable to calculate the effect of time on the risk of dementia. Fourth, our analysis could not categorize dementia subtypes (e.g., AD and vascular dementia) with SRDs due to a lack of data. Fifth, a significant heterogeneity was observed in the meta-analysis. Different study designs, geographical regions, and confounding factors may have contributed to this heterogeneity. Therefore, a random effects model was utilized because it could estimate the mean value of the distribution of effect sizes for heterogeneous populations. We also conducted subgroup analyses based on the study design and methodological quality (**Supplementary Table S3**). Finally, a small number of studies assessed OA and dementia risks that prevented the application of funnel plots to measure actual publication bias. Furthermore, we applied the trim-and-fill method to check the variance of the observed and adjusted values (67).

## Conclusion

This systematic review and meta-analysis found that SRDs, such as OA, SLE, and SS, were associated with an increased risk of dementia. However, no significant association was observed between RA and dementia.

## Data availability statement

The original contributions presented in the study are included in the article/**Supplementary Material**. Further inquiries can be directed to the corresponding author.

## Author contributions

Y-CW and W-MK proposed the research idea and wrote the draft. M-SL and C-CW performed the analysis. AP-HH supported and contributed the literature review. Y-CW, M-SL, and W-MK helped revise the manuscript. M-SL and AP-HH provided clinical suggestions. Y-CW and W-MK supported data analysis and prepared the manuscript for submission. All authors contributed to the article and approved the submitted version.

## Conflict of interest

The authors declare that the research was conducted in the absence of any commercial or financial relationships that could be construed as a potential conflict of interest.

## Publisher’s note

All claims expressed in this article are solely those of the authors and do not necessarily represent those of their affiliated organizations, or those of the publisher, the editors and the reviewers. Any product that may be evaluated in this article, or claim that may be made by its manufacturer, is not guaranteed or endorsed by the publisher.
