# Supplementary material for: Association between systemic rheumatic diseases and dementia risk: A meta-analysis
Source: Front Immunol. 2022 Nov 9;13:1054246. doi: 10.3389/fimmu.2022.1054246 (PMC9682025; doi:10.3389/fimmu.2022.1054246)
Supplement: Supplementary file 1 [file Table_1.docx]

**Supplementary Table S1**. Search strategies.

| Keywords | MESH terms |
| --- | --- |
| Systemic rheumatic diseases  Osteoarthritis  Rheumatoid arthritis  Systemic lupus erythematosus  Sjogren’s syndrome | ("systemic"[All Fields] OR "systemically"[All Fields] OR "systemics"[All Fields]) AND ("rheumatic diseases"[MeSH Terms] OR ("rheumatic"[All Fields] AND "diseases"[All Fields]) OR "rheumatic diseases"[All Fields]) OR  "osteoarthritis"[MeSH Terms] OR "osteoarthritis"[All Fields] OR "osteoarthritides"[All Fields]) OR  "arthritis, rheumatoid"[MeSH Terms] OR ("arthritis"[All Fields] AND "rheumatoid"[All Fields]) OR "rheumatoid arthritis"[All Fields] OR ("rheumatoid"[All Fields] AND "arthritis"[All Fields]) OR  "lupus erythematosus, systemic"[MeSH Terms] OR ("lupus"[All Fields] AND "erythematosus"[All Fields] AND "systemic"[All Fields]) OR "systemic lupus erythematosus"[All Fields] OR ("systemic"[All Fields] AND "lupus"[All Fields] AND "erythematosus"[All Fields]) OR  "sjogren s syndrome"[MeSH Terms] OR ("sjogren s"[All Fields] AND "syndrome"[All Fields]) OR "sjogren s syndrome"[All Fields] OR ("sjogren"[All Fields] AND "syndrome"[All Fields]) OR "sjogren syndrome"[All Fields] |
| Dementia  Alzheimer’s disease  Cognitive impairment | "dementia"[MeSH Terms] OR "dementia"[All Fields] OR "dementias"[All Fields] OR "dementia s"[All Fields] OR  "alzheimer disease"[MeSH Terms] OR ("alzheimer"[All Fields] AND "disease"[All Fields]) OR "alzheimer disease"[All Fields] OR  "alzheime s"[All Fields] OR "alzheimer disease"[MeSH Terms] OR ("alzheimer"[All Fields] AND "disease"[All Fields]) OR "alzheimer disease"[All Fields] OR "alzheimer"[All Fields] OR "alzheimers"[All Fields] OR "alzheimer s"[All Fields] OR "alzheimers s"[All Fields] OR  "cognitive dysfunction"[MeSH Terms] OR ("cognitive"[All Fields] AND "dysfunction"[All Fields]) OR "cognitive dysfunction"[All Fields] OR ("cognitive"[All Fields] AND "impairment"[All Fields]) OR "cognitive impairment"[All Fields] |
