# Supplementary material for: Association between systemic rheumatic diseases and dementia risk: A meta-analysis
Source: Front Immunol. 2022 Nov 9;13:1054246. doi: 10.3389/fimmu.2022.1054246 (PMC9682025; doi:10.3389/fimmu.2022.1054246)
Supplement: Supplementary file 2 [file Table_2.docx]

**Supplementary Table S2**. Characteristics of included studies and Newcastle-Ottawa Scale (NOS) evaluation.

| Source | Country | Study design | Follow-up | Adjusted variables | NOS |
| --- | --- | --- | --- | --- | --- |
| Swain 2021 | UK | Prospective cohort | 5 | Adjusted for age, gender, BMI, alcohol use, smoking, multimorbidity count and index year. | 9 |
|  |  | Retrospective case-control | 5 | Adjusted for age, gender, BMI, alcohol use, smoking, multimorbidity count and index year. | 9 |
| Park 2021 | Korea | Retrospective case-control | NR | adjusted for age, sex, income, residence city size, comorbidities | 9 |
| Innes-2020 | USA | Cohort | 2-3 | Sex, age, race/ethnicity, education, income, supplemental insurance, marital status, region, smoking status, BMI, chronic physical health conditions (including RA, lupus), history of stroke, TBI, joint, back, and neck pain;  neuropathic pain, headache, migraine | 9 |
| Min 2020 | Korea | Case-control | NR | Age, sex, income, region of residence, hypertension, diabetes, and dyslipidemia histories, ischemic heart disease, cerebral stroke, depression, and Sjogren’s syndrome histories | 9 |
|  |  | Cohort | NR | Age, sex, income, region of residence, hypertension, diabetes, and dyslipidemia histories, ischemic heart disease, cerebral stroke, depression, and Sjogren’s syndrome histories | 9 |
| Huang 2019 | Taiwan | Case-control | NR | age, gender, and all comorbidities | 8 |
| Chen 2019 | Taiwan | Case-control | 12 | age, gender, and all comorbidities | 8 |
| Chen 2018 | Taiwan | Case-control | NR | age, gender, and all comorbidities | 8 |
| Li 2018 | Sweden | Case-control | 1.8 | age, gender, and all comorbidities | 7 |
| Wang 2018 | Taiwan | Case-control | NR | Age, gender, and all comorbidities | 7 |
| Lin 2018 | Taiwan | Cohort | 5 | Age, gender, and all comorbidities | 8 |
| Kao 2016 | Taiwan | Case-control | NR | Age, gender, and all comorbidities | 8 |
| Lin 2016 | Taiwan | Cohort | NR | Age, gender, and all comorbidities | 7 |
| Huang 2015 | Taiwan | Cohort | 4 | Age, gender, and all comorbidities | 8 |
| Lu 2014 | Taiwan | Cohort | 5 | Age, gender, and all comorbidities | 7 |
| Veeranki 2017 | Mexico | Cohort | 21 |  | 6 |
| Wotton 2017 | UK | Case-control | ~5 | Age, gender, and all comorbidities | 8 |
| Kang 2010 | Taiwan | Case-control | NR | Age, gender, and all comorbidities | 7 |
