# Supplementary material for: Association between systemic rheumatic diseases and dementia risk: A meta-analysis
Source: Front Immunol. 2022 Nov 9;13:1054246. doi: 10.3389/fimmu.2022.1054246 (PMC9682025; doi:10.3389/fimmu.2022.1054246)
Supplement: Supplementary file 3 [file Table_3.docx]

**Supplementary Table S3.** Subgroup analyses based on study design and methodological quality.

|  |  | **Effect size** | | **Heterogeneity** | | | |
| --- | --- | --- | --- | --- | --- | --- | --- |
|  | No. of study | RR with 95% CI | P-value | Q | I^2^ | τ^2^ | p-value |
| **Study design** | | | | | | | |
| Cohort | 7 | 1.32 (1.14 – 1.52) | <0.001 | 103.38 | 90.32 | 0.03 | <0.001 |
| Case-control | 10 | 1.13 (1.05 – 1.22) | <0.001 | 260.85 | 92.71 | 0.02 | <0.001 |
| **Methodological quality** | | | | | | | |
| Low | 6 | 1.24 (1.08 – 1.43) | 0.001 | 27.15 | 66.85 | 0.02 | 0.001 |
| High | 11 | 1.15 (1.06 – 1.26) | 0.001 | 533.53 | 96.06 | 0.03 | <0.001 |
